# Immunohistochemical Expression of Vascular Endothelial Growth Factor (VEGF) in Primary Canine Mast Cell Tumors and Related Regional Lymph Node Metastasis

**DOI:** 10.3390/ani15020283

**Published:** 2025-01-20

**Authors:** Alice Corrêa Rassele, Isabella Oliveira Almeida, Maylla Garschagen Gava, Pedro Antônio Bronhara Pimentel, Antonio Giuliano, Felipe Augusto Ruiz Sueiro, Ayisa Rodrigues de Oliveira, Andrigo Barboza de Nardi, Rodrigo dos Santos Horta

**Affiliations:** 1 Department of Veterinary Medicine and Surgery, Agricultural and Veterinary Sciences, Universidade Estadual Paulista, Jaboticabal 14884-900, Brazil; alicerassele@yahoo.com.br; 2 Department of Veterinary Medicine and Surgery, Veterinary School, Universidade Federal de Minas Gerais, Belo Horizonte 31310-250, Brazil; isabellaoalm@gmail.com (I.O.A.); pedrobpimentel@gmail.com (P.A.B.P.); ayisa.rodrigues@gmail.com (A.R.d.O.); 3 Maylla Gava Patologia Veterinaria, Praia das Gaivotas, Vila Velha 29102-571, Brazil; mayllagava@hotmail.com; 4 Department of Veterinary Clinical Science, Jockey Club College of Veterinary Medicine, City University of Hong Kong, Kowloon, Hong Kong; agiulian@cityu.edu.hk; 5 CityU Veterinary Medical Centre, City University of Hong Kong, Kowloon, Hong Kong; 6 VETPAT, Campinas 13070-070, Brazil; felipesueiro@hotmail.com

**Keywords:** neoplasm, dog, prognostic, treatment, dissemination

## Abstract

Vascular endothelial growth factor (VEGF) is an important regulator of angiogenesis and has been identified as an autocrine growth factor for neoplastic cells of several tumors. The aim of this study was to evaluate VEGF expression in canine mast cell tumors and their respective lymph node metastases. For this purpose, 28 patients with mast cell tumors and confirmed regional lymph node metastasis were selected. The samples were submitted for analysis with immunohistochemistry. VEGF was expressed in 14/28 patients (50%) and 35.7% of primary MCTs (10/28), and only 14.3% expressed VEGF in both the primary tumor and its respective metastasis (4/28), with fair agreement (Κ = 0.250). There was a weak correlation between VEGF and tumor size (*p* = 0.016, rs = 0.045). In this cohort, survival time was correlated with Kiupel grade, mitotic index, tumor necrosis, tumor location, and systemic treatment. VEGF immunolabeling had no influence on survival; however, patients with positive expression may benefit from specific therapy.

## Simple Summary

VEGF (vascular endothelial growth factor) is a cellular growth factor involved in angiogenesis. It has been detected in many canine neoplasms and it is recognized as a potential target for drug therapy. This study evaluated the immunohistochemical expression of VEGF in canine mast cell tumors and their respective lymph node metastases in order to determine the concordance of this expression and the correlation with other prognostic factors and patient survival. Only 14.3% expressed VEGF both on the primary tumor and its respective metastasis, with fair agreement (Κ = 0.250). VEGF immunolabeling had no influence on survival time.

## 1. Introduction

The incidence of neoplasms in companion animals has been increasing as a result of longer life expectancy [1]. In most species, neoplasms involving mast cells are uncommon; however, in dogs, mast cell tumors (MCTs) represent 20.9 to 22.4% of cutaneous neoplasms [1,2,3,4,5].

MCTs exhibit wide variation in biological behavior, ranging from indolent to highly invasive and metastatic [1,2,6,7,8,9]. Tumor grade is considered one of the most reliable prognostic factors [10,11,12]; however, there is great variability in the biological behavior of MCTs with similar histological features and grade, often complicating the prognosis and decision-making process [3,4,7,9]. Several studies have investigated histological, immunohistochemical, and genetic features to improve prognostication and treatment [10,11,12]. Despite some improvement, the biological behavior of some MCTs remains difficult to predict.

Vascular endothelial growth factor (VEGF) and its receptors (VEGFRs) are described as regulators of angiogenesis in various organs [13,14,15]. VEGF is a mitogenic factor for the endothelium of arteries, veins, and lymphatic vessels [16,17]. This factor is responsible for the proliferation of vascular endothelial cells and migration to form capillary and larger-caliber vessels [18]. Angiogenesis is initiated when VEGF binds to VEGFR-1 receptors, encoded by the Flt-1 gene (c-FMS-like tyrosine kinase), and VEGFR-2, encoded by the Flk-1 gene (fetal liver kinase) [19]. Studies indicate that, in addition to endothelial cells, Flt-1 and Flk-1 receptors are also located in the glandular epithelium and in the human endometrial stroma [20]. VEGF also interacts with a family of co-receptors, the neuropilins NP1 and NP2, increasing the affinity for VEGFR-1 and -2 [21,22].

VEGF has been identified as both a mediator of angiogenesis and an autocrine growth regulator in cells with different degrees of malignancy [13,14,15,23]. In certain canine neoplasms, VEGF and VEGFRs were also found overexpressed, becoming potential targets for drug therapies [24]. The VEGF expression in canine primary MCTs and respective metastases has not been previously reported. Thus, a deeper understanding of the role of VEGF and other growth factors in tumor angiogenesis and growth may contribute to the development of new therapies.

This study aimed to evaluate the immunohistochemical expression of VEGF in primary MCTs and lymph node metastasis to compare the concordance of expression and evaluate the correlation with other prognostic factors and the survival time of these patients.

## 2. Materials and Methods

### 2.1. Case Selection and Tissue Sampling

Twenty-eight patients with confirmed histopathological diagnosis of MCTs and metastasis to the regional lymph node were retrospectively collected from veterinary practices in the state of Espirito Santo/ES (Brazil) between April 2020 and December 2021.

All patients were classified according to the proposed amendment to the WHO system of clinical staging of cutaneous and subcutaneous MCTs in dogs by Horta et al. (2018) classification [12] as stage III (single tumor with regional lymph node involvement).

Patients with more than one nodule, multicentric or subcutaneous presentation, or with insufficient clinical history and follow-up were not included. Information regarding breed, age, sex, location of the primary tumor, affected lymph node, size, and tumor surface were collected. Clinical follow-ups were performed at veterinary clinics or by telephone with the oncologist responsible for patient care.

### 2.2. Histopathological Assessment

The edges of the primary tumor fragment were painted with India ink and used for microscopic evaluation of the surgical margins, as described by Stromberg and Meuten (2017) [25] M1 for infiltrated margin (focal or diffuse), M2 for close margin (<2 mm), M3 for clean margins between 2 and 5 mm, and M4 for clean margins larger than 5 mm. Longitudinal sections of 2 mm were made for paraffin embedding for the primary tumor and lymph node (including the perilymph node fat). The slides were prepared with a microtome and stained with toluidine blue and hematoxylin-eosin (HE). Microscopic evaluation of primary tumors included evaluation of surgical margins and mitotic figure count (in 10 high-power fields at 400× fields—FN22—2.37 mm^2^). Grading was performed according to histomorphological criteria defined by two different studies: Patnaik et al. (1984) and Kiupel et al. (2011) [6,9]. The evaluation of the lymph nodes followed the criteria proposed by Weishaar et al. (2014) [26], considering the level of involvement of the lymph node by the mast cell tumor in three categories: suspicious (HN1), initial metastasis (HN2) or evident (HN3).

### 2.3. Immunohistochemical Assessment

Immunohistochemistry was performed using antibodies against VEGF (clone VG1, DakoCytomation). Tumors were sectioned into 3 μm thick slices and mounted on slides coated with 3-amino-propyltriethoxysilane. Slides were deparaffinized and rehydrated in a graded ethanol series.

The treatment was performed with preheated EDTA, pH 5.6. Antibodies were diluted in antibody diluent at a ratio of 1:200. VEGF expression was measured in all samples and staining was evaluated according to Table 1, where staining was scored as ‘0’ (no staining detected in tumor cells); ‘+/’ (<10% of the tumor area showed positive staining); ‘+’ (10–25% stained); ‘++’ (>25–50% stained); or ‘+++’ (>51% stained). Expression was defined as negative when cytoplasmic staining was less than 10% and positive when stained at a value greater than or equal to 10% adapted from [27].

### 2.4. Statistical Analysis

The concordance of VEGF expression in the primary tumor and lymph node was assessed by Cohen’s Kappa test. For the interpretation, results ranging from slight to almost perfect agreement were considered, according to the table with the agreement values below (Table 2) [28,29].

Pearson’s correlation was also performed, considered significant with *p* < 0.05 and R < 0.3 indicating a weak correlation, 0.3 to 0.7 a moderate correlation, and >0.7 a strong correlation. Survival time was estimated using the Kaplan–Meier curve and evaluated from surgery to death, censoring those who were lost during follow-up or died for reasons unrelated to the tumor. Comparisons according to VEGF expression, as well as Patnaik and Kiupel grading, Weishaar lymph node classification, and use of systemic adjuvant therapy, were performed using the log rank test. Differences with *p* < 0.05 were considered significant. All statistical analyses were performed in GraphPadPrism v. 6.02.

## 3. Results

### 3.1. Clinical Data

Among the 28 dogs included in this study, 42.8% (16/28) were females and 57.1% (12/28) were males. Ages ranged between 4 and 14 years, with an average of 9 years. Mixed-breed dogs represented 25% (7/28) of patients. French Bulldogs and Labrador Retrievers accounted for 10.7% (3/28), while Pitbulls and Boxers represented 7.1% (2/28) each. The remaining breeds, Beagle, Dogo Argentino, Lhasa Apso, Maltese, Pinscher, Poodle, Miniature Schnauzer, Sharpei, Shih-tzu, Dachshund, and Yorkshire Terrier each represented 3.6% each (1/28).

The location of primary tumors varied, with 25% (7/28) found in the limbs, 21.4% (6/28) in the scrotum, and 10.7% (3/28) in the labial region, while the breasts, thorax, back, and digits corresponded to 7.1% each (2/28). The abdomen, base of the ear, interdigital, and prepuce regions each represented 3.6% (1/28). Regarding to the characteristics of the tumor surface, 25% (7/28) had erythema, 53.6% (15/28) were alopecic, 21.4% (6/28) were ulcerated, and necrosis was observed in 28.6% (8/28) of the tumors. Tumor size ranged between 1 and 7 cm.

Paraneoplastic syndromes were observed in 17.8% of patients (5/28), with edema and erythema being the most common, present in 4/5. Anemia, thrombocytopenia, hemorrhage, gastritis, enteritis, and emesis were noted in 2/5.

The lymph nodes were evaluated based on the expected anatomical lymphatic drainage of the excised primary MCT. Of the lymph nodes analyzed, 39.3% (11/28) corresponded to the superficial inguinal lymph node, while axillary and popliteal lymph nodes represented 17.8% (5/28) each, and mandibular and prescapular lymph nodes each represented 14.3% (4/28) (Table 3).

Regarding the treatments performed on patients, 75% (21/28) underwent neoadjuvant cytoreductive therapy with the use of corticosteroids (oral prednisolone before surgery) and 25% (7/28) did not.

In terms of adjuvant chemotherapy, 78.6% received some form of medical treatment, while 21.4% (6/28) did not. Of those who received adjuvant treatment, 27.3% (6/22) received vinblastine monotherapy, 9.1% (2/22) lomustine monotherapy, 9.1% (2/22) a tyrosine kinase inhibitor (Toceranib phosphate) in monotherapy, and 4.5% (1/22) cyclophosphamide in monotherapy. Among combination treatments, 22.8% (5/22) received vinblastine with lomustine, 13.6% (3/22) received vinblastine with toceranib phosphate, 4.5% (1/22) received lomustine with toceranib phosphate, and 9.1% (2/22) received vinblastine and lomustine with toceranib phosphate.

Local recurrence occurred in 10.7% (3/28) of the patients, and those three died due to disease progression. Five patients died but did not present local recurrence, totaling 28.6% (8/28) of the deaths. After surgical excision, 21.4% (6/28) of the patients did not undergo any chemotherapy protocol and among these, 2/6 had local recurrence and died of tumor-related causes, and 1/3 died of tumor-related causes without local recurrence.

### 3.2. Histopathological and Immunohistochemical Analysis

Among the histopathological evaluations of the MCTs in this study, it was observed that 53.6% (15/28) were grade II/low grade, 25% (7/28) were grade II/high grade, and 21.4% (6/28) were grade III/high grade. HN1 lymph nodes represented 7.1% (2/28); HN2 corresponded to 46.5% (13/28), among which 76.9% were low grade; and 23.1% were high grade. The remaining 46.5% (13/28) were classified as HN3, with 76.9% being high grade and 23.1% being low grade (Table 4).

**Figure 1:**
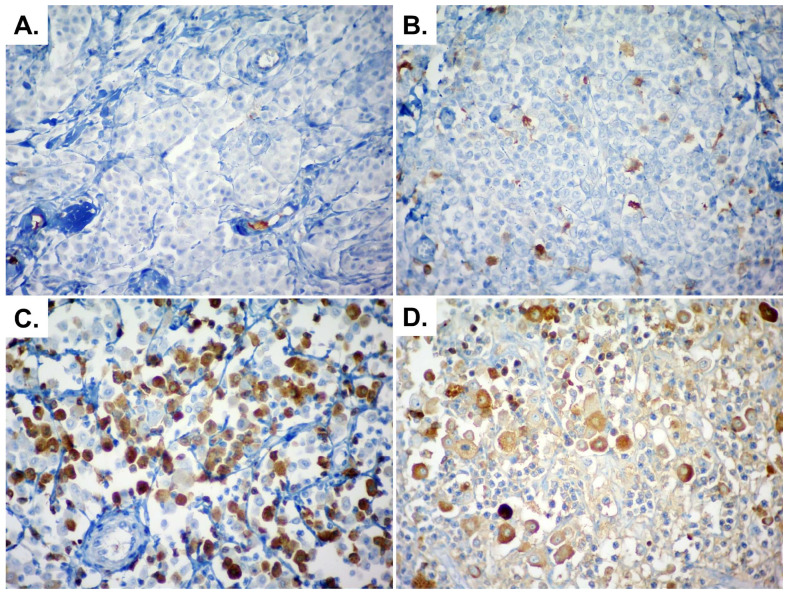
VEGF immunolabeling on canine mast cell tumor and nodal metastasis. Immunolabeling in less than 10% of neoplastic cells (+/ and −; negative) on primary tumor (**A**) and lymph node metastasis (**B**) and in more than 10% of neoplastic cells on primary tumor (+++; positive) (**C**) and lymph node metastasis (++; positive) (**D**). Hematoxylin with DAB immunolabeling, 400×.

VEGF immunoexpression is shown in Figure 1. VEGF positivity was observed in 14/28 patients (50%). Specifically,35.8% expressed VEGF only in the primary MCTs (10/28), while 14.2% (4/28) in both the primary tumor and its respective metastasis (4/28). No cases demonstrated VEGF exclusively in the metastasis. There was a fair level of agreement in VEGF expression between primary tumors and metastases (Κ = 0.251).

A moderate correlation was observed between Kiupel grading system and both the number of mitotic figures and the classification of lymph node metastasis. Similarly, a moderate correlation was observed between the number of mitotic figures in the primary tumor and the lymph node metastasis classification. Tumor necrosis and ulceration demonstrated a moderate correlation with Kiupel and Patnik grading schemes, as well as the number of mitotic figures and lymph node classification. Regarding VEGF expression, a weak correlation was noted between its expression in the primary tumor and tumor size. Paraneoplastic syndromes were correlated with recurrence and patient death.

**Figure 2:**
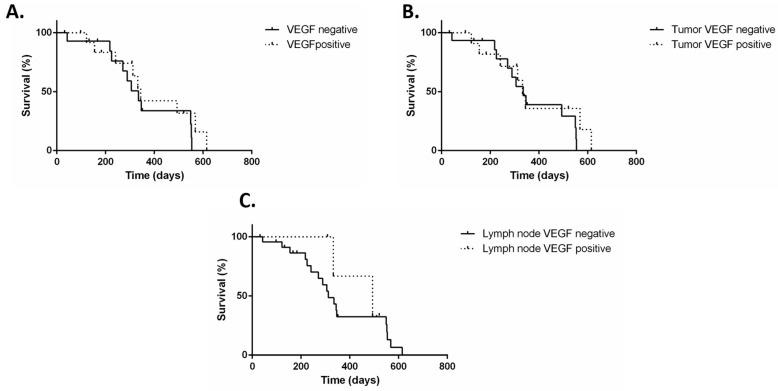
Kaplan–Meier survival curves of 28 dogs with mast cell tumors according to immunoexpression. (**A**) VEGF immunoexpression on the primary tumor or lymph node (median not reached; *p* = 0.6799); (**B**) VEGF immunoexpression only in the primary tumor (median reached only for VEGF negative group at 521 days; *p* = 0.8328); (**C**) VEGF immunoexpression only in nodal metastasis (median not reached; *p* = 0.6458).

Survival curves are shown in Figure 2. Regarding the histopathological evaluation of the tumor, the median survival was reached only for grade III MCTs at 144.5, with a difference in the log-rank comparison (*p* < 0.0001). A similar result was observed in the Kiupel grading scheme and the median was reached only for high-grade MCTs at 175 days (*p* < 0.0001). In the evaluation of the nodal status, the median survival was reached only for the HN3 group at 521 days.

Due to the limited number ofHN1 cases (n = 2), the median survival time for this group was not calculated. The median survival was not reached for the HN2 group, and no significant difference was found in the survival curves based on lymph node classification (*p* = 0.1485). Regarding VEGF expression, the median survival time was not reached in either the VEGF-positive or VEGF-negative groups, and there was no significant difference in the logrank test (*p* = 0.6799). When the primary tumors were evaluated individually based on VEGF expression, the median survival was reached only in the VEGF-negative group (521 days), but no significant difference was observed in the survival curves (*p* = 0.8328). In the evaluation of the lymph nodes, the median survival times were not reached and no significant difference were found (*p* = 0.6458). When considering treatment, the median survival was reached only in patients who did not undergo systemic therapy (175 days) (*p* < 0.0001) (Table 5).

## 4. Discussion

Canine MCTs present a highly variable biological behavior, and several factors are currently used for prognostic evaluation [1,2,6,7,8,30]. Histopathological grading is considered the most reliable method for predicting the biological behavior of canine MCTs. Previously, Patnaik system [6] was the most commonly used grading method. However, the predominance of grade II MCTs and the low interobserver agreement in various studies decreased confidence in this classification system.

Kiupel et al. [9] assessed the effectiveness of the histopathological grading proposed by Patnaik and found that interobserver agreement was 75% for grade III MCTs but less than 64% for grades I and II. These results revealed a relevant difference between the pathologists, indicating the subjectivity of this grading scheme. In response, Kiupel et. al. [9] proposed a a two-tier grading system as an alternative.

In this study, both grading schemes were applied. A higher frequency of grade II/low-grade tumors was observed, followed by grade II/high-grade, with grade III/high-grade tumors being the least frequent. Well-differentiated grade I tumors, which have a low mitotic index, often exhibit good behavior, with the vast majority cured by surgical excision, and fewer than 10% forming metastases [6,26]. Grade I MCTs were not observed in this study.

Intermediate tumors have variable biological behavior, with metastatic rates from 5% to 22% [6,26]. Although typically classified as Kiupel low-grade, other prognostic factors should be considered [12]. In a study conducted by Stefanello et al. [31] involving 368 dogs, metastasis was found in regional lymph nodes in 15% of MCTs classified as low-grade. Bae et al. [32] evaluated 121 Kiupel low-grade MCTs associated with biologically aggressive behavior, including nodal or distant metastases.

In the present study, 78.6% of the tumors evaluated were grade II, of which 68.2% were classified as low-grade and 31.8% as high-grade, consistent with findings in the literature. Predicting MCTs biological behavior remains challenging. Therefore, it is currently recommended to evaluate canine MCTs using both grading schemes, along with the mitotic index and other clinical and molecular prognostic factors [12].

Sentinel lymph node removal in dogs with MCTs is well-documented, and the presence of metastasis is recognized as an important prognostic factor. A study conducted by Marconato et al. [33] demonstrated that dogs with grade II tumors benefit from lymph node extirpation; 152 dogs were evaluated, among which 81 underwent lymphadenectomy and confirmation of metastasis by histopathology, while 71 did not undergo resection, but the metastasis was confirmed by cytology. Univariate analysis revealed a significantly higher risk of developing local, nodal, or distant recurrence in the group that did not undergo resection (*p* < 0.001). Multivariate analysis also showed an increased risk of tumor progression and death (*p* < 0.001). Similar findings were obtained in a study conducted by Chalfon et al. [34], which evaluated 49 animals with grade II and high-grade tumors, 31 of whom underwent lymphadenectomy. The median time to progression and overall survival time were significantly lower in dogs that did not undergo lymphadenectomy. In multivariate analysis, the absence of lymphadenectomy was associated with a higher risk of overall tumor progression, nodal progression, and tumor-related death.

Another study focused on dogs with grade I MCTs undergoing regional lymph node extirpation was conducted by Sabattini et al. [35]. This study evaluated 64 dogs with low-grade/grade I tumors, of which 29 underwent lymphadenectomy, while the others were part of the control group. The survival curves showed a tendency towards an improved progression free survival time in the group that underwent nodal extirpation (*p* = 0.058), and disease progression was significantly more frequent in the control group (*p* = 0.028).

These findings suggest that lymphadenectomy of the MCTs’ regional lymph node, regardless of tumor grade, may improve survival time. Furthermore, the histopathological classification of the metastases can provide valuable prognostic information. The histopathological evaluation proposed by Weishaar et al. [26] assists in the classification of mast cells present in suspected lymph nodes of dogs with MCTs. Patients with lymph nodes classified as HN0/HN1 were associated with a better prognosis when compared to those classified as HN2/HN3.

In this study, HN2 lymph nodes were more frequently observed, followed by HN3; however, no significant differences in the survival curves were found, likely due to the influence of systemic chemotherapy. It is worth noting that the median survival was only reached in dogs that did not receive systemic chemotherapy.

The expression of VEGF in cancer has been extensively studied and has already been reported in some neoplasms of dogs and cats [36,37,38,39,40]. Millanta et al. [39] reported a correlation between increased VEGF expression and loss of tumor differentiation in mammary neoplasms of dogs and cats. However, the quantitative VEGF expression was not associated with clinical staging or survival. In dogs with canine soft tissue sarcoma, high VEGF staining (more than 75% of neoplastic cells) was significantly associated with increased local recurrence and reduced survival time (*p* < 0.001). Conversely, high expression of decorin (type III—labeled in the stroma saturating the entire tumor), an antiangiogenic factor, was associated with better outcomes, although only VEGF was significantly associated with survival in the multivariate analysis [41].

Multiple studies indicate that VEGF and its receptors are expressed in certain malignant tumors, including solid and hematopoietic neoplasms [42,43,44,45,46], and function as an autocrine growth factor [23,47,48]. VEGF not only promotes angiogenesis but may also correlate with the aggressiveness of neoplasms [49,50,51,52]. Interestingly, it has been observed that normal mast cells can express and release VEGF, indicating that mast cell-derived VEGF may play a role in the regulation of angiogenesis [53,54,55]. Thus, the role of VEGF in MCTs remains poorly understood and warrants investigation.

In the present study, only 14.8% of patients expressed VEGF in both primary tumor and metastasis, with fair agreement. Additionally, a weak correlation was found between VEGF expression and tumor size. However, no difference in survival time was noted between VEGF-positive primary tumors and metastasis and those without VEGF expression in either site. Similar results have been reported. In a study carried out by Rebuzzi et al. [56], they described variable VEGF expression and receptor scores in canine MCTs across different grades. Therefore, MCTs are likely do not utilize VEGF as an autocrine growth regulator. Similarly, Amorim et al. [55] reported no significant differences in the proportion or intensity of VEGF immunostaining between tumor grades, suggesting that VEGF alone is not a reliable biomarker for malignancy.

In contrast, a recent study conducted by Melo et al. [27] shows that strong and moderate expressions of VEGF may serve as a negative prognostic factor, as intense VEGF staining was significantly associated with decreased survival time (*p* = 0.025). However, no correlation was found between the expressions of the factor with the development of metastases or compromise of surgical margins. To date, no study of VEGF expression in MCT regional lymph node metastases has been carried out. In the present study, we observed no correlation between VEGF expression and metastasis development, tumor grade, other clinical prognostic factors, or survival time.

VEGF likely does not represent a relevant prognostic factor in canine MCT and high VEGF-expression may not be essential in local or distant progression. However, we did not evaluate the VEGFR and it is possible that overexpression of the VEGFR plays a more critical role than VEGF. It could become a therapeutic target in an attempt to control tumor growth and its metastases.

In human medicine, antiangiogenic agents have been employed in the treatment of different types of cancer, mainly based on the inhibition of pro-angiogenic tumor signaling pathways, particularly the VEGF/VEGFR axis [56]. Drugs such as bevacizumab (Avastin^®^), sunitinib (Sutent^®^, SU11248), and sorafenib (Nexavar^®^, BAY 43-9006) exhibit antiangiogenic activity by inhibiting VEGF signaling through VEGF ligand-receptor blockade [57].

Anti-angiogenic therapy is also used in veterinary medicine. Metronomic chemotherapy with low-dose cyclophosphamide upregulates the antiangiogenic protein thrombospondin-1, with consequent apoptosis of endothelial cells and suppression of tumor growth [58]. Similarly, toceranib, initially developed as an antiangiogenic compound like sunitinib, inhibits VEGFR and PDGFRβ, while also exerting direct anti-tumor effects by targeting KITr [59]. VEGFR overexpression in canine MCT, despite VEGF levels, may highlight the relevance of these treatments in a subset of patients.

This study has some limitations, including its retrospective nature and the small sample size. While the removed lymph node was the regional one, most likely to receive the lymphatic flow from the primary tumor, sentinel lymph node mapping using lymphographic techniques was not performed. Therefore, it cannot be confirmed that the removed lymph nodes were indeed the sentinel nodes. Additionally, the interpretation of the classification of the staining was subjective and reliant on the pathologist, with no quantitative measurement. Further studies with larger case numbers and evaluation of other angiogenic factors like VEGFR, PDGFR, and microvascular density, could provide a deeper understanding of angiogenesis in MCT progression and metastasis.

## 5. Conclusions

Immunohistochemical analysis of VEGF in canine MCTs can be easily performed; however, it is not routinely used due to its unclear clinical relevance. In our study, no correlation was found between VEGF expression in the primary tumor and its associated regional lymph node metastasis with patient survival. As such, the analysis of this factor does not seem to contribute to prognostic assessment. Nevertheless, evaluating VEGF expression may still aid in tailoring individualized therapeutic approach for patients; however, more studies are required to confirm its potential clinical utility.

## Figures and Tables

**Table 1:** VEGF assessment method.

Staining	Score	Result
0	−	Negative
<10%	+/	Negative
10–25%	+	Positive
25–50%	++	Positive
>51%	+++	Positive

**Table 2:** Cohen’s Kappa test.

Values	Agreement
0.01–0.20	Slight
0.21–0.40	Fair
0.41–0.60	Moderate
0.61–0.80	Substantial
0.81–1.00	Almost Perfect

**Table 3:** Histological grade and VEGF immunoexpression in 28 dogs with mast cell tumor and lymph node metastasis.

Tumor Region	Affected Lymph Node	Patnaik	Kiupel	Metastasis in Lymph Node	VEGF in the Primary Tumor	VEGF in the Lymph Node	Survival (Months)
Abdomen	Inguinal	II	High grade	HN3	P	N	10
Ear	Mandibular	II	Low grade	HN2	N	N	10
Scrotum	Inguinal	III	High grade	HN2	P	P	1
Scrotum	Inguinal	II	Low grade	HN2	P	N	11
Scrotum	Inguinal	III	High grade	HN3	N	N	5
Scrotum	Inguinal	II	High grade	HN3	P	P	11
Scrotum	Inguinal	III	High grade	HN3	N	N	9
Scrotum	Inguinal	II	High grade	HN3	P	N	6
Digit	Superficial Cervical	III	High grade	HN3	P	N	3
Digit	Superficial Cervical	II	High grade	HN2	N	N	4
Back	Axillary	II	Low grade	HN2	P	N	5
Back	Axillary	II	Low grade	HN3	P	N	8
Interdigit	Popliteal	II	Low grade	HN2	N	N	7
Labial Region	Submandibular	II	High grade	HN3	N	N	11
Labial Region	Submandibular	III	High grade	HN3	P	N	4
Labial Region	Submandibular	II	Low grade	HN3	N	N	11
Breast	Axillary	II	Low grade	HN2	P	P	11
Breast	Inguinal	II	High grade	HN3	P	P	10
Pelvic Limb	Popliteal	II	High grade	HN2	N	N	7
Pelvic Limb	Popliteal	II	Low grade	HN1	N	N	11
Pelvic Limb	Inguinal	II	Low grade	HN1	N	N	18
Pelvic Limb	Popliteal	II	Low grade	HN2	N	N	18
Pelvic Limb	Inguinal	II	Low grade	HN3	N	N	18
Thoracic Limb	Superficial Cervical	II	Low grade	HN2	N	N	9
Thoracic Limb	Superfical Cervical	III	High grade	HN3	P	N	16
Prepuce	Inguinal	II	Low grade	HN2	N	N	10
Thoracic	Axillary	II	Low grade	HN2	P	N	20
Thoracic	Axillary	II	Low grade	HN2	P	N	18

P: positive; N: negative.

**Table 4:** VEGF expression in primary mast cell tumors and respective nodal metastases according to the histopathological grade of the primary tumor using Patnik and Kiupel schemes.

VEGF Expression in Tumor and Metastasis	Grade II/Low Grade	Grade II/High Grade	Grade III/High Grade
Tumor P/Metastasis P	1/4 (25%)	2/4 (50%)	1/4 (25%)
Tumor P/Metastasis N	5/10 (50%)	2/10 (20%)	3/10 (30%)
Tumor N/Metastasis P	-	-	-
Tumor N/Metastasis N	9/14 (64.3%)	3/14 (21.4%)	2/14 (14.3%)

P stands for positive immunolabeling and N for negative immunolabeling.

**Table 5:** Pearson correlation with respective *p* and *r* values in 28 dogs with cutaneous mast cell tumors.

	*p* < 0.05	R Correlation
**Kiupel grade**		
Tumor Necrosis	0.004	0.520
Tumor Ulceration	0.004	0.520
Patnaik’s Graduation	0.001	0.560
Mitotic Index	0.0001	0.665
Lymph Node Classification	0.001	0.567
Paraneoplastic Syndromes	0.006	−0.500
**Patnaik grade**		
Tumor Necrosis	0.0002	0.633
Ulceration	0.019	0.440
Paraneoplastic Syndromes	0.019	−0.438
**Mitotic Index**		
Tumor Necrosis	0.010	0.478
Tumor Ulceration	0.006	0.503
Lymph Node Classification	0.003	0.530
**Lymph Node Classification**		
Tumor Necrosis	0.007	0.493
Tumor Ulceration	0.007	0.493
**Tumor Ulceration**		
Paraneoplastic Syndromes	0.003	−0.530
**Tumor Location**		
Tumor Size	0.010	−0.475
**VEGF in the tumor**		
Tumor Size	0.016	0.258
**Paraneoplastic Syndromes**		
Recurrence	0.018	0.441
**Death related to mast cell tumor**		
Kiupel grade	0.004	−0.520
Tumor Size	0.038	−0.392
Recurrence	0.002	0.547
**Survival**		
Kiupel grade	0.031	−0.406
Mitotic index	0.047	0.378
Tumor necrosis	0.025	−0.422
Tumor location	0.005	−0.509
Systemic Treatment	0.017	−0.445
**Chemotherapy**		
Breed	0.0003	0.623
Size	0.012	−0.225

## Data Availability

The original data presented in the study are openly available in the Unesp repository at https://repositorio.unesp.br/server/api/core/bitstreams/9fb7da3d-5337-45d1-89cc-01d68d71136e/content. accessed on 10 November 2024.
