# Correction: Corrêa Rassele et al. Immunohistochemical Expression of Vascular Endothelial Growth Factor (VEGF) in Primary Canine Mast Cell Tumors and Related Regional Lymph Node Metastasis. *Animals* 2025, *15*, 283

**DOI:** 10.3390/ani15101391

**Published:** 2025-05-12

**Authors:** Alice Corrêa Rassele, Isabella Oliveira Almeida, Maylla Garschagen Gava, Pedro Antônio Bronhara Pimentel, Antonio Giuliano, Felipe Augusto Ruiz Sueiro, Ayisa Rodrigues de Oliveira, Andrigo Barboza de Nardi, Rodrigo dos Santos Horta

**Affiliations:** 1 Department of Veterinary Medicine and Surgery, Agricultural and Veterinary Sciences, Universidade Estadual Paulista, Jaboticabal 14884-900, Brazil; alicerassele@yahoo.com.br; 2 Department of Veterinary Medicine and Surgery, Veterinary School, Universidade Federal de Minas Gerais, Belo Horizonte 31310-250, Brazil; isabellaoalm@gmail.com (I.O.A.); pedrobpimentel@gmail.com (P.A.B.P.); ayisa.rodrigues@gmail.com (A.R.d.O.); 3 Maylla Gava Patologia Veterinaria, Praia das Gaivotas, Vila Velha 29102-571, Brazil; mayllagava@hotmail.com; 4 Department of Veterinary Clinical Science, Jockey Club College of Veterinary Medicine, City University of Hong Kong, Kowloon, Hong Kong; agiulian@cityu.edu.hk; 5 CityU Veterinary Medical Centre, City University of Hong Kong, Kowloon, Hong Kong; 6 VETPAT, Campinas 13070-070, Brazil; felipesueiro@hotmail.com

## Newly Added Table

In the original publication [1], Table 5, which refers to the Results was missing; Table 5 is shown below.

## Update Corresponding Author and Author Contribution

Andrigo Barboza de Nardi was included as a corresponding author as he also participated on Conceptualization, Methodology, Formal Analysis, Writing—Original Draft Preparation, Writing—Review and Editing, Supervision. The study was also performed in collaboration with Universidade Estadual Paulista (UNESP). Andrigo Barboza de Nardi’s telephone number is Tel.: +55-16-3209-7247.

## Text Correction

There was an error in the original publication. On Discussion, paragraph 12, it is stated that “In the present study, only 21.4% of patients expressed VEGF in both primary tumor and metastasis, with fair agreement.” The percentage presented must be replaced by 14.8% as in the results previously presented: “In the present study, only 14.8% of patients expressed VEGF in both primary tumor and metastasis, with fair agreement.”

The authors state that the scientific conclusions are unaffected. This correction was approved by the Academic Editor. The original publication has also been updated.

## Figures and Tables

**Table 5:** Pearson correlation with respective *p* and *r* values in 28 dogs with cutaneous mast cell tumors.

	*p* < 0.05	R Correlation
**Kiupel grade**		
Tumor Necrosis	0.004	0.520
Tumor Ulceration	0.004	0.520
Patnaik’s Graduation	0.001	0.560
Mitotic Index	0.0001	0.665
Lymph Node Classification	0.001	0.567
Paraneoplastic Syndromes	0.006	−0.500
**Patnaik grade**		
Tumor Necrosis	0.0002	0.633
Ulceration	0.019	0.440
Paraneoplastic Syndromes	0.019	−0.438
**Mitotic Index**		
Tumor Necrosis	0.010	0.478
Tumor Ulceration	0.006	0.503
Lymph Node Classification	0.003	0.530
**Lymph Node Classification**		
Tumor Necrosis	0.007	0.493
Tumor Ulceration	0.007	0.493
**Tumor Ulceration**		
Paraneoplastic Syndromes	0.003	−0.530
**Tumor Location**		
Tumor Size	0.010	−0.475
**VEGF in the tumor**		
Tumor Size	0.016	0.258
**Paraneoplastic Syndromes**		
Recurrence	0.018	0.441
**Death related to mast cell tumor**		
Kiupel grade	0.004	−0.520
Tumor Size	0.038	−0.392
Recurrence	0.002	0.547
**Survival**		
Kiupel grade	0.031	−0.406
Mitotic index	0.047	0.378
Tumor necrosis	0.025	−0.422
Tumor location	0.005	−0.509
Systemic Treatment	0.017	−0.445
**Chemotherapy**		
Breed	0.0003	0.623
Size	0.012	−0.225
